# Burden of anemia in patients with osteoarthritis and rheumatoid arthritis in French secondary care

**DOI:** 10.1186/1471-2318-10-59

**Published:** 2010-08-26

**Authors:** Gergana Zlateva, Ruth Diazaraque, Muriel Viala-Danten, Liviu Niculescu

**Affiliations:** 1 Pfizer Inc, New York, NY, USA; 2 Pfizer Inc, Walton Oaks, UK; 3 Mapi Values, Lyon, France

## Abstract

**Background:**

Arthritic disorders can be the cause of hospitalizations, especially among individuals 60 years and older. The objective of this study is to investigate associations between health care resource utilization in arthritis patients with and without concomitant anemia in a secondary care setting in France.

**Methods:**

This retrospective cohort study utilized data on secondary care activity in 2001 from the Programme de Médicalisation des Systèmes d'Information database. Two cohorts were defined using ICD-10 codes: patients with an arthritis diagnosis with a concomitant diagnosis of anemia; and arthritis patients without anemia. Health care resource utilization for both populations was analyzed separately in public and private hospitals. Study outcomes were compared between the cohorts using standard bivariate and multivariable methods.

**Results:**

There were 300,865 hospitalizations for patients with arthritis only, and 2,744 for those with concomitant anemia. Over 70% of patients with concomitant anemia were in public hospitals, compared with 53.5% of arthritis-only patients. Arthritis patients without anemia were younger than those with concomitant anemia (mean age 66.7 vs 74.6, public hospitals; 67.1 vs 72.2, private hospitals). Patients with concomitant anemia/arthritis only had a mean length of stay of 11.91 (SD 14.07)/8.04 (SD 9.93) days in public hospitals, and 10.68 (SD 10.16)/9.83 (SD 7.76) days in private hospitals. After adjusting for confounders, the mean (95% CI) additional length of stay for arthritis patients with concomitant anemia, compared with those with arthritis only, was 1.56 (1.14-1.98) days in public and 0.69 (0.22-1.16) days in private hospitals. Costs per hospitalization were €;480 (227-734) greater for arthritis patients with anemia in public hospitals, and €;30 (-113-52) less in private hospitals, than for arthritis-only patients.

**Conclusions:**

Arthritis patients with concomitant anemia have a longer length of stay, undergo more procedures, and have higher hospitalization costs than nonanemic arthritis patients in public hospitals in France. In private hospitals, concomitant anemia was associated with modest increases in length of stay and number of procedures; however, this did not translate into higher costs. Such evidence of anemia-related health care utilization and costs can be considered as a proxy for the clinical significance of anemia.

## Background

Arthritis and other rheumatic conditions are a significant public health issue, and are estimated to affect more than 21% of adults [1]. Osteoarthritis (OA), the most common form of arthritis, is a major cause of disability [2,3] and presents a significant burden to health care providers [4]. In approximately 10% of the world's population aged 60 years or more, OA-related joint pathology causes significant clinical problems [5]. Rheumatoid arthritis (RA) is less prevalent but is associated with high medical expenses because of the requirement for continuous treatment to slow disease progression, and a high incidence of joint replacements [6]. Hospitalization represents a significant component of the costs associated with arthritis [7,8].

Anemia, defined by the World Health Organization [9] as a hemoglobin concentration below 12 g/dl in women and 13 g/dl in men, is common in people with arthritis. Anemia is associated with increased morbidity, length of hospitalization, and cost of care delivery [10]. In RA, it is estimated that 30-60% of patients are anemic [11-14]. One of the most frequent causes of anemia in RA patients is "iron deficiency anemia," which can result from gastrointestinal (GI) bleeding related to nonsteroidal anti-inflammatory drug (NSAID) use [15,16]. "Anemia of chronic disease," which does not usually respond to iron supplementation, is another major cause of anemia in patients with RA [17,18]. In a study of 225 patients with RA, anemia of chronic disease accounted for 77% and iron deficiency anemia for 23% of observed anemia [14]. There are few data on the prevalence of anemia in patients with OA, although the prevalence of both conditions is known to increase with age [5,19-21]. Sex also appears to influence disease prevalence; OA appears to affect more women than men, while current estimates suggest that women are up to three times more likely to develop RA than men [22]. Women are also at a greater risk of becoming anemic than men, particularly during menstruation or pregnancy, when iron requirements are increased [23].

Information on the impact of anemia in arthritic populations is also limited, although there is evidence that anemic RA patients have more severe arthritic disease than nonanemic patients [14,24,25]. Studies in other populations have demonstrated that the clinical impact of anemia is substantial: for example in chronically ill patients, anemia has been associated with an increased risk of mortality and morbidity while also having a negative impact on quality of life [18]; Anand and colleagues also demonstrated that anemic patients with chronic heart failure have greater disease severity and have a higher risk of hospitalization or death [26]. Adverse outcomes related to anemia may be of particular importance in the elderly, in whom anemia (and arthritic disease) is common [19]. Penninx et al. have associated anemia in elderly populations with an increased risk of hospitalization (adjusted relative risk 1.27 [95% confidence interval [CI] 1.12-1.45]) and mortality (relative risk 1.61 [95% CI 1.34-1.93]) [27], as well as disability, poor physical performance, and decreased muscle strength [28-30]. In community-dwelling older women, even mild anemia and low-normal hemoglobin levels have been identified as independent risk factors for frailty: compared with a hemoglobin concentration of 13.5 g/dl, adjusted odds for frailty of 11.5 g/dl and 12 g/dl were 1.9 (95% CI 1.1-3.4) and 1.5 (95% CI 1.0-2.1) [31].

Anemia has also been linked to increased health care costs and resource utilization [32], with direct medical costs for anemic patients with comorbid conditions up to twice those for nonanemic patients with the same comorbid condition [33,34]. However, data on anemia-associated resource use and cost in people with arthritis remain very sparse.

The objective of this study was to assess differences in health resource utilization patterns among arthritis (OA and RA) patients with concomitant anemia compared with those without anemia. We analyzed data from French hospital admissions to test the hypothesis that arthritis patients with concomitant anemia are associated with more health care resource use than nonanemic arthritis patients.

## Methods

### Study design

This retrospective cohort study utilized data on secondary care in France from the Programme de Médicalisation des Systèmes d'Information (PMSI) database. For public and private hospitals, two cohorts were identified from the hospitalizations that occurred during the 2001 calendar year: hospitalizations where there was a primary/secondary diagnosis of arthritis without anemia; and hospitalizations where there was both a primary/secondary diagnosis of arthritis and a primary/secondary diagnosis of anemia. Thus, both cohorts were comprised of arthritis patients, but differed in the presence or absence of anemia diagnosis, respectively. The cohorts were compared for the following measures of hospital resource utilization: length of stay, number of procedures, and mean total cost.

The PMSI database covers more than 90% of private and public hospital activity in France and is used by government and regional health authorities as a tool to provide hospital activity indicators for allocating annual budget and forecasting medical needs and resources. Even though the PMSI database is not exhaustive of French hospitals, it guarantees a standardized collection of data that allows the unbiased identification of cases and controls for epidemiological studies. For each hospital stay, the PMSI database includes information on the patient's age, sex, and postal region; their primary, secondary, and related diagnoses; procedures undertaken; and length of stay. Use of PMSI to assess the epidemiological and economic burden of illness is recommended by the French guidelines for health economic evaluation [35].

Patients admitted to a hospital were classified by primary diagnosis and then allocated a randomized code to maintain anonymity. Access to patient information thereafter was available only at the hospital admission or hospital stay level. The two study cohorts were identified from the PMSI database using *International Statistical Classification of Diseases and Related Health Problems, 10th Revision *(*ICD-10*) codes [36]. Arthritis, defined as RA and/or OA, included seropositive RA (*ICD-10 *code M05), other RA (M06), polyarthrosis (M15), coxarthrosis/hip arthrosis (M16), gonarthrosis/knee arthrosis (M17), arthrosis of first carpometacarpal joint (M18), and other arthrosis (M19). A diagnosis of anemia included iron deficiency anemia (D50.0, D50.1, D50.8, D50.9), vitamin B_12 _deficiency anemia (D51.0-D51.3, D51.8, D51.9), folate deficiency anemia (D52.0, D52.1, D52.8, D52.9), other nutritional anemias (D53.0-D53.2, D53.8, D53.9), and acquired hemolytic anemias (D59.0-D59.6, D59.8, D59.9). Patients with alpha and beta thalassemia were not included.

### Costs

Health care resource utilization for length of stay, number of procedures, and total cost of stay in public and private hospitals was analyzed for both study populations. Costs were assessed from the health care system perspective, and were calculated according to the French Diagnosis-Related Group (DRG) system. This system classifies every hospital patient into one of several hundred DRG groups that are intended to be clinically meaningful and homogenous with respect to resource use. The DRG assignment was recorded on the PMSI dataset for each hospitalization.

Due to differences in the reference costs and financing systems of public and private hospitals in France, reference cost information was not equivalent in the two sectors and, therefore, was not directly comparable. For individual public hospitals in each region, the mean cost per DRG is calculated and expressed in a synthetic index, called the ISA (Index Synthétique d'Activité); the number of ISA points represents an index of hospital productivity. For public hospitals, the average ISA value is, in part, calculated by dividing the "short-term stays" activity budget by the number of ISA points for this activity. However; for private hospitals or clinics, the ISA point value is calculated from the expenses reimbursed by the public health insurance fund (Social Security Sick Funds) to these hospitals. Furthermore, because the financing systems differ, costing for public and private hospitals has to be performed and interpreted separately.

For public hospitals (which represent three quarters of all hospitalized patients), two published lists of reference costs were used to calculate costs for hospital stays for the study populations [37]. The reference costs, calculated from accountability data from a subgroup of 40 public hospitals, were given per DRG for an average length of stay for each DRG, allowing calculation of unit costs per day for each DRG. Total costs of admissions in each of the two study populations were calculated by summing the product of the length of stay for each admission and the appropriate DRG-related unit cost per day for each admission.

The reference unit costs per hospital stay per DRG comprised the following: medical and paramedical care (salaries of clinicians, nurses, and other medical staff); pharmaceuticals and drugs; anesthesia (including operation suite); laboratory tests and procedures; intensive care (medical staff salaries, pharmaceuticals, amortization, maintenance, and medical logistics); logistics (amortization and medical material maintenance, medical logistics, food service, central laundry, hospital management); structure (amortization of building and facilities and committed fixed costs); and reanimation costs (costs associated with acute care; similar but not equivalent to intensive care in the US). The average total cost per stay in public hospitals was broken down into these categories.

For private hospitals we used the reference cost lists, calculated on all medical fees reimbursed to patients by the Social Security Sick Funds [38], to calculate total costs per stay. Because of the method used for its development, the list of reference costs for private hospitals did not include assessment of detailed items of costs, such as in the public sector. Only total costs of stay per DRG, DRG-related procedures, and medical care corresponding to an invoice sent to the Social Security Sick Fund for reimbursement, were available. Thus, part of the fixed costs, maintenance, logistics, and salaries are not included in the private total costs per stay.

### Statistical analysis

Our primary analysis was to describe the resource utilization of hospitalized patients with arthritis in the two study groups (arthritis with anemia cohort and arthritis-only cohort). The description of resource utilization data was done by their means and 95% CIs. Five confounders of the impact of anemia on resource utilization were identified: sex (male, female), age (0-59, 60-69, 70-79, 80+ years), type of primary diagnosis of arthritis (presence or absence of each of the following diagnoses: M05, M06, M15, M16, M17, M19), type of secondary/associated diagnosis of arthritis (presence or absence of each of the following diagnoses: M05, M06, M15, M16, M17, M19), and number of associated diagnoses/comorbidities (none, 1-2, 3-4, 5-6, 6+).

In order to eliminate the possible effect of these confounders on resource utilization when comparing arthritis with anemia and arthritis-only cohorts, we randomly generated "matched-control" samples from the arthritis without anemia population based on the five confounders. Simple random sampling was performed in the arthritis-only cohort stratified by confounders. The stratum sampling rates were specified as being equal to the actual percentages observed in the arthritis with anemia cohort. Five separate replications of this random process were performed leading to five independent matched-samples. This approach allowed obtaining five control samples (no anemia) comparable to the population with anemia in terms of sociodemographics, diagnostics, and comorbidities. Then the resource utilization data were described and compared between "matched-control" samples and the arthritis with anemia population by their means and 95% CIs.

To further confirm these results, we also conducted multivariable analyses on the entire study sample, including the confounding variables and the variable "anemia yes/no." We described and compared the adjusted resource utilization for the two study groups (arthritis with anemia cohort and arthritis-only cohort) using analysis of covariance. All data processing and analyses were performed using SAS software (Statistical Analysis System, version 8. 2, SAS Institute, Inc., Cary, NC).

## Results

Results for public and private hospitals are presented separately, because of the inherent differences in care structure and cost capture in the two settings.

### Public hospitals

#### Number of hospitalizations

In public hospitals in 2001 there were 161,121 hospitalizations for the arthritis without anemia population and 1,941 hospitalizations for the arthritis with anemia population (Table 1). Overall, the prevalence of anemia with arthritis in public hospitals was 1.2% (1941/163,062).

**Table 1:** Number of hospitalizations and characteristics of study populations

	Public		Private	
	
	Arthritis without anemia	Arthritis with anemia	Arthritis without anemia	Arthritis with anemia
Number of hospitalizations, n (%)	**161,121 (98.8)**	**1,941 (1.2)**	139,783 (99.4)	803 (0.6)

Mean age, y (SD)	**66.7 (15.1)**	**74.6 (13.8)**	67.1 (12.4)	72.2 (12.4)
Sex, n (%)				
Female	**108,514 (67.4)**	**1,484 (76.5)**	84,808 (60.7)	601 (74.8)
Male	**52,605 (32.7)**	**457 (23.5)**	54,970 (39.3)	202 (25.2)
Number of associated diagnoses Mean (SD)	**2.5 (2.5)**	**5.8 (5.8)**	2.2 (2.4)	5.3 (3.3)
Median	**2.0**	**5.0**	2.0	5.0

#### Characteristics of study population

The mean age of the arthritis without anemia population was slightly lower than that of the arthritis with anemia population; 66.7 years and 74.6 years, respectively (Table 1). In the arthritis with anemia population, three quarters of arthritis patients were female, whereas in the arthritis without anemia population, female patients comprised approximately two thirds of the population (Table 1).

Rheumatoid arthritis (nonseropositive RA; 18.5%), gonarthrosis (11.1%), arthrosis (10.3%), and other primary coxarthrosis (9.8%) represented the most frequently recorded diagnoses in the arthritis without anemia population in public hospitals. The most frequent diagnoses recorded in the hospitalizations of patients with concomitant arthritis and anemia were iron deficiency anemia (17.5%), iron deficiency anemia (secondary to blood loss; 12.7%), and RA (nonseropositive RA; 11.6%), followed by other iron deficiency anemias (7.4%), polyarthrosis (6.4%), and arthrosis (6%).

On average, patients with arthritis and anemia had a greater number of associated diagnoses (in addition to arthritis and/or anemia), compared with arthritis patients without anemia (5.8 vs 2.5, respectively; Table 1). Essential (primary) hypertension was the most common associated diagnosis (comorbidity) in the arthritis with anemia and arthritis without anemia populations, accounting for 5.9% and 7.5% of associated diagnoses, respectively. In the arthritis with anemia population, the most common comorbidities in public hospitals, other than essential hypertension, were blood transfusion (2.4%; as defined by *ICD-10*), atrial fibrillation and flutter (1.4%), diaphragmatic hernia (without obstruction or gangrene; 1.3%), ulcer of the lower limb (1.1%), and elevated erythrocyte sedimentation rate (1.1%). Other than essential hypertension, the most frequently reported comorbidities in the arthritis without anemia population included obesity (1.5%), type 2 diabetes (without complication; 1.4%), other chemotherapy or medical care (1.3%), and atrial fibrillation or flutter (1.2%).

#### Hospital treatment of study population, descriptive results

Arthritis patients with concomitant anemia stayed significantly longer in public hospitals (mean 11.9 [95% CI 11.28-12.54] days) than those without anemia (mean 8.0 [95% CI 7.99-8.09] days) (Table 2). The mean number of procedures performed on patients in public hospitals was also significantly greater for those with concomitant anemia (3.7 [95% CI 3.48-3.92] procedures) than for patients without anemia (2.6 [95% CI 2.58-2.62] procedures; Table 2).

**Table 2:** Resource utilization, descriptive results^a^

	Public		Private	
	**Arthritis without anemia**	**Arthritis with anemia**	**Arthritis without anemia**	**Arthritis with anemia**
	**n = 161,121**	**n = 1,941**	**n = 139,783**	**n = 803**

Mean length of stay, days (SD)	**8.0 (9.9)**	**11.9 (14.1)**	9.8 (7.8)	10.7 (10.2)
Missing, n	**4,403**	**26**	2,087	25

Mean number of associated procedures (SD)	**2.6 (3.2)**	**3.7 (4.9)**	4.3 (2.8)	4.8 (3.7)

Mean total cost, € (SD)	**4,190 (5,940)**	**5,870 (9,433)**	3,380 (2,038)	2,700 (2,100)
Missing, n	**4,499**	**29**	188	3

^a^Due to differences in the reference costs and financing systems of public and private hospitals in France, reference cost information was not equivalent in the two sectors and, therefore, was not directly comparable.

The types of procedures performed were mainly related to general anesthesia management/use of recovery room services, cardiovascular function monitoring (e.g., electrocardiography), and thoracic or abdominal investigations. In arthritic patients without anemia, procedures generally focused on the arthritis diagnosis, with major surgeries undertaken, such as hip and knee replacements, comprising 7.2% of all procedures in public hospitals. In patients with concomitant anemia, procedures were more focused on identifying the cause of anemia and its monitoring, with hip and knee replacements each representing <1% of procedures performed in public hospitals.

**Figure 1:**
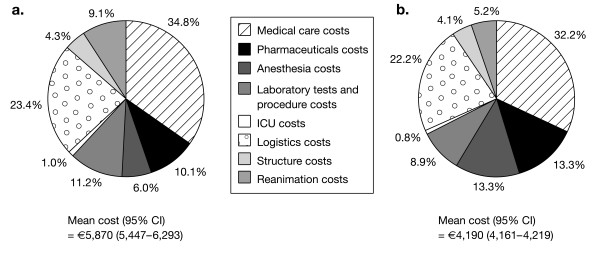
**Cost breakdown in public hospitals ^a^among arthritis patients (a) with anemia or (b) without anemia**. ^a^Not available for private hospitals. ICU, intensive care unit.

Overall, the mean cost per hospitalization in public hospitals for the arthritis and anemia population was €;5,870 (95% CI 5,447-6,293) and €;4,190 (95% CI 4,161-4,219) for the arthritis without anemia population (Table 2). In the public hospital sector, anesthesia accounted for a greater proportion of the total cost of stay for arthritic patients without concomitant anemia than those with anemia (13.3 vs 6.0%, Figure 1). However, the concomitant anemia population had a higher proportion of cost attributable to laboratory tests and procedures (11.2 vs 8.9%) and reanimation care (9.1 vs 5.2%).

#### Hospital treatment of study population, comparative results

Tables 3, 4 and 5 show the results of the univariable analysis conducted on the "matched-control" samples, and the multivariable analysis conducted on the whole population for length of stay, number of procedures, and total cost, adjusting for sex, age, type of primary arthritis diagnosis, type of secondary arthritis diagnoses, and number of associated diagnoses. The results of the univariable analysis conducted on the matched-control samples are consistent with the results of the multivariable analysis for each outcome.

**Table 3:** Differences between arthritis and anemia versus arthritis without anemia in length of stay, adjusted for age, sex, type of primary diagnosis of arthritis, type of secondary/associated diagnosis of arthritis, and number of associated diagnoses

		Public	Private
		
		Difference, days (95% CI)	Difference, days (95% CI)
Univariable analysis	Matched sample #1	**0.91 (0.01 to 1.81)**	0.35 (-0.67 to 1.37)
	
	Matched sample #2	**0.68 (**-**0.20 to 1.56)**	0.47 (-0.55 to 1.49)
	
	Matched sample #3	**1.30 (0.47 to 2.13)**	0.79 (-0.19 to 1.77)
	
	Matched sample #4	**1.00 (0.11 to 1.89)**	0.29 (-0.70 to 1.28)
	
	Matched sample #5	**1.13 (0.28 to 1.98)**	0.03 (-1.07 to 1.13)

Multivariable analysis	Whole population	**1.56 (1.14 to 1.98)**	0.69 (0.22 to 1.16)

Legend: Matched samples of arthritis patients with anemia and without anemia were identified based on five confounders: Sample #1 - gender (male, female); Sample #2 - age (0–59, 60–69, 70–79, 80+); Sample #3 - type of primary diagnosis of arthritis; Sample #4  - type of secondary/associated diagnosis of arthritis; Sample #5 - number of associated diagnoses/comorbidities (none, 1–2, 3–4, 5–6, 6+).

**Table 4:** Differences between arthritis and anemia versus arthritis without anemia in number of procedures, adjusted for age, sex, type of primary diagnosis of arthritis, type of secondary/associated diagnosis of arthritis, and number of associated diagnoses

		Public	Private
		
		Difference, no of procedures (95% CI)	Difference, no of procedures (95% CI)
Univariable analysis	Matched sample #1	**0.58 (0.29 to 0.87)**	0.17 (-0.16 to 0.50)
	
	Matched sample #2	**0.48 (0.20 to 0.76)**	-0.12 (-0.46 to 0.22)
	
	Matched sample #3	**0.45 (0.17 to 0.73)**	0.14 (-0.20 to 0.48)
	
	Matched sample #4	**0.44 (0.16 to 0.72)**	0.02 (-0.32 to 0.36)
	
	Matched sample #5	**0.45 (0.18 to 0.72)**	0.17 (-0.17 to 0.51)

Multivariable analysis	Whole population	**0.52 (0.38 to 0.65)**	0.08 (-0.11 to 0.27)

Legend: Matched samples of arthritis patients with anemia and without anemia were identified based on five confounders: Sample #1 - gender (male, female); Sample #2 - age (0–59, 60–69, 70–79, 80+); Sample #3 - type of primary diagnosis of arthritis; Sample #4  - type of secondary/associated diagnosis of arthritis; Sample #5 - number of associated diagnoses/comorbidities (none, 1–2, 3–4, 5–6, 6+).

**Table 5:** Differences between arthritis and anemia versus arthritis without anemia in total cost, adjusted for age, sex, type of primary diagnosis of arthritis, type of secondary/associated diagnosis of arthritis, and number of associated diagnoses

		Public	Private
		
		Difference, € (95% CI)	Difference, €; (95% CI)
Univariable analysis	Matched sample #1	**280 (-320 to 880)**	26 (-180 to 232)
	
	Matched sample #2	**144 (-416 to 704)**	28 (-178 to 234)
	
	Matched sample #3	**371 (-193 to 935)**	-11 (-219 to 197)
	
	Matched sample #4	**167 (-451 to 785)**	-21 (-228 to 186)
	
	Matched sample #5	**374 (-174 to 922)**	-58 (-263 to 147)

Multivariable analysis	Whole population	**480 (227 to 734)**	-30 (-113 to 52)

Legend: Matched samples of arthritis patients with anemia and without anemia were identified based on five confounders: Sample #1 - gender (male, female); Sample #2 - age (0–59, 60–69, 70–79, 80+); Sample #3 - type of primary diagnosis of arthritis; Sample #4  - type of secondary/associated diagnosis of arthritis; Sample #5 - number of associated diagnoses/comorbidities (none, 1–2, 3–4, 5–6, 6+).

After adjustment using multivariable analysis on the whole population, in public hospitals the mean length of stay for arthritis patients with concomitant anemia, compared with those with arthritis only, was significantly higher, with an additional 1.56 days (95% CI 1.14-1.98; Table 3) of stay. Arthritic patients with anemia also underwent significantly more procedures than those without anemia (0.52 additional procedures; 95% CI 0.38-0.65; Table 4). The additional length of stay and number of procedures attributable to anemia in public hospitals represents 20% of the mean length of stay and number of procedures for nonanemic arthritis patients. Furthermore, the total cost per hospitalization for arthritis patients with anemia was €;480 greater (95% CI 227-734) than for patients with arthritis without anemia (Table 5). The adjusted difference in costs per hospitalization between the two populations (€;480 [95% CI 227-734]), represents 11% of the mean cost expenditure for nonanemic arthritis patients.

### Private hospitals

#### Number of hospitalizations

Overall, in 2001 there were 139,783 private hospitalizations for the arthritis without anemia population and 803 hospitalizations for the arthritis with anemia population. Overall, the prevalence of anemia with arthritis in private hospitals was 0.6% (n = 803/140,586).

#### Characteristics of study population

As for public hospitalizations, the mean age of the arthritis with anemia population was slightly greater than that of the arthritis without anemia population, 72.2 years and 67.1 years, respectively (Table 1). Again, female patients comprised approximately three quarters of the arthritis with anemia population and 61% of the arthritis without anemia population (Table 1).

In terms of the primary or secondary diagnoses in the arthritis without anemia population, coxarthrosis (other primary; 20.4%), gonarthrosis (other primary; 17.4%), gonarthrosis (7.5%), and arthrosis (7.2%) were the most frequently reported. In patients with arthritis and anemia, the most common diagnoses were iron deficiency anemia (secondary to blood loss; 18.6%), iron deficiency anemia (17.6%), RA (nonseropositive RA; 9.1%), and coxarthrosis (other primary; 7.8%).

Private hospital patients with arthritis and concomitant anemia also had, on average, a greater number of associated diagnoses (in addition to arthritis and/or anemia), than arthritis patients without anemia (5.3 vs 2.2, respectively; Table 1). Again, similar for public hospitalizations, the most common associated diagnosis was essential (primary) hypertension, accounting for 8.8% and 13.3% of associated diagnoses in the arthritis with anemia and arthritis without anemia populations, respectively. In arthritic patients with anemia, the most common associated diagnoses other than essential hypertension were diaphragmatic hernia (without obstruction or gangrene; 2.8%), diverticular distension of the large intestine (without perforation or abscess; 2.1%), blood transfusion (without reported diagnosis; 2.1%), and pure hypercholesterolemia (2.0%). In arthritic patients without anemia, other than hypertension, the most common associated diagnoses were pure hypercholesterolemia (3.0%), obesity (2.5%), type 2 diabetes (without complication; 2.1%), and varicose veins of the lower extremities (without ulcer or inflammation; 1.5%).

#### Hospital treatment of study population, descriptive results

In private hospitals, the mean unadjusted length of stay for arthritis patients with anemia was 10.7 (95% CI 9.97-11.39) days, versus 9.8 (95% CI 9.79-9.87) days for arthritis patients without anemia (Table 2). In private hospitals, patients underwent more procedures than in public hospitals (mean 4.8 [95% CI 4.57-5.07] for anemic arthritis patients and 4.3 [95% CI 4.28-4.30] for nonanemic patients; Table 2).

As for public hospitals, the types of procedures performed were mainly related to general anesthesia management/use of recovery room services, cardiovascular function monitoring (e.g., electrocardiography), and thoracic or abdominal investigations. In arthritic patients without anemia, procedures generally focused on the arthritis diagnosis, with major surgeries undertaken, such as hip and knee replacements, comprising 12% of all procedures in private hospitals. In patients with concomitant anemia, procedures were more focused on identifying the cause of anemia and its monitoring, with hip and knee replacements (combined) representing 7% (hip and knee combined) of all procedures in private hospitals.

In private hospitals, the mean cost per hospitalization for the arthritis and anemia population was €;2,700 (95% CI 2,554-2,846) compared with €;3,380 (95% CI 3,369-3,391) for the arthritis without anemia population (Table 2).

#### Hospital treatment of study population, comparative results

Tables 3, 4 and 5 show the results of the univariable and multivariable analysis conducted on the matched-control and whole population samples for length of stay, number of procedures, and total cost, adjusting for sex, age, type of primary arthritis diagnosis, type and number of secondary arthritis diagnoses, and number of associated diagnoses. The results of the univariable analysis conducted on the matched-control samples are consistent with the results of the multivariable analysis for each outcome.

In private hospitals, after adjustment using multivariable analysis on the whole population, the differences between the anemic and nonanemic populations in length of stay and number of procedures were more modest than those seen in public hospitals (0.69 days [95% CI 0.22-1.16] and 0.08 procedures [95% CI -0.11 to 0.27], respectively; Tables 3 and 4). The total cost per private hospitalization was €;30 less (95% CI -113 to 52) for arthritis patients with anemia than for patients with arthritis who did not have concomitant anemia (Table 5).

In both public and private hospitals, the combined total economic burden of all hospitalizations for arthritis patients with and without anemia was €;13.6 million and €;1.15 billion, respectively. Of the €;13.6 million, €;907,590 was attributable to anemia.

## Discussion

This retrospective cross-sectional study assessed the 2001 hospital health care utilization and costs related to anemia in arthritis patients in France. The study showed that, in public hospitals, arthritis patients with concomitant anemia use more health care resources than those without anemia. After adjustment for confounders, the length of stay and number of procedures attributable to anemia were greater in public than in private hospitals. Although it was not possible to directly compare the two populations due to the way in which these costs were calculated, the cost of stay per hospitalization in public hospitals was greater in patients with concomitant anemia than in those without anemia, while in private hospitals anemia was associated with a modest decrease in the total cost of stay.

To our knowledge, there are no previous studies looking specifically at the impact of anemia on health care resource use in hospitalized populations in Europe. However, studies assessing overall annual health care utilization have shown that anemia is associated with increased resource use and costs [32-34]. In a US study based on administrative claims data for anemic adults with RA or one of five other comorbid conditions, utilization of key medical services was significantly higher (*P *< 0.001) for anemic than nonanemic patients [34]. In addition, anemic patients had higher care (including inpatient) costs than nonanemic patients with the same comorbid condition, and inpatient and outpatient costs more than double those for nonanemic patients after adjustment for confounders. Another US study identified a difference in direct costs between anemic and nonanemic RA patients of more than $7,000 per year, and showed that anemia also impacts indirect health care costs [33]. Penninx et al. [27] examined the relationship of anemia with death and hospitalization outcomes in a community-based sample of older people. They identified a significant association between anemia and subsequent mortality and hospitalization (relative risk 1.61 [95% CI 1.34-1.93] and relative risk 1.27 [95% CI 1.12-1.45], respectively) and, consistent with the results from the French public hospital sector in the current study, found that people with anemia who were hospitalized had a significantly longer length of stay than nonanemic patients (25.0 vs 13.7, respectively, *P *< 0.001) [27].

The use of a national database representative of hospitalizations across France, in conjunction with the large size of the two study populations involved (303,648 hospitalizations, representing 1.6% of all admissions to French hospitals in 2001) adds weight to the findings regarding the impact of anemia in an arthritic population. In addition, the adjustment for key factors that could influence health care resource use, particularly the primary/secondary diagnosis and number of associated diagnoses, helps guard against the possibility that anemia is simply a marker for greater morbidity and severity of underlying disease. Indeed, analysis of the number and types of procedures performed in anemic and nonanemic patients suggests that, in this study, the anemic population has less severe arthritic disease than the nonanemic population.

However, this study also has several limitations. First, as the analyzed data is derived from retrospective analysis of medical claims, the findings do not denote causality, but rather focus on identifying temporal association among patient outcomes. We are thus identifying association, not causality, between anemia and increased resource utilization. Second, care must be taken when using DRGs as these are sometimes re-coded for billing purposes and may, therefore, no longer accurately reflect the actual final diagnosis [39]. Indeed, even though DRGs are intended to be clinically consistent with respect to resource use, the calculated costs remain an average estimate that does not reflect the heterogeneity among severity of diseases included in the DRG. Third, the prevalence of anemia in the hospitalized arthritis patients (0.9%) seems low compared with previous estimates of 30-60% prevalence of anemia in people with RA [11-14], which may have biased the outcome. However, most patients in this analysis were OA patients, in whom the prevalence of anemia may not be as high. Moreover, our study did not include patients with "anemia of chronic disease," a common type of anemia in people with RA [18,17]. Another possible reason for the low percentage of anemic patients in this study is that anemia is often presented and treated in the primary care setting, and thus may have been successfully treated in a proportion of the study population before they were hospitalized. A fourth limitation is that the database identifies "admission" and not "patient," so some patients may have been counted twice (if they had two admissions for their arthritis or anemia during the year). Additionally, the database identifies only admissions for which the anemia or arthritis required a specific treatment of the patient during his or her admission. Arthritic/anemic patients who were admitted to the hospital for a reason other than their arthritis/anemia were not identified. Last, anemia was measured as the hemoglobin threshold and did not take into account its severity and clinical impact.

It is interesting that the results of this study show an anemia-attributable increase in resource utilization and cost in public but not in private hospitals. A small increase was observed in the length of stay of anemic versus nonanemic patients in the private sector, but this did not translate into increased costs, probably because of the less detailed way in which costs are reported for this sector. In private hospitals, because only total costs of stay per DRG (comprising procedures 0 and medical care corresponding to an invoice sent to the Social Security Sick Fund for reimbursement) were available, part of the fixed costs such as, maintenance, logistics, and salaries, were not included in the private total costs per stay 0.

In private hospitals, the anemia-related increase in length of stay was less than half that observed for public hospitals, and there was no difference in the number of procedures undergone by anemic and nonanemic patients. It is possible that the presence of anemia has a greater impact in patients who are generally more unwell than in those who are not as sick. In this study, diagnoses of patients in private hospitals were generally less severe (for example, there were fewer RA patients and the mean number of associated diagnoses was significantly lower) than those in public hospitals, reflecting the different characteristics of the two sectors: public hospitals, which are mostly teaching hospitals, offer more technical and innovative procedures and allow treatment of more severe cases than private centers, which are more sensitive to activity profitability. Furthermore, differences in financial incentives, corresponding to an increased stay in either public or private hospitals, may have also influenced these findings, though this is beyond the current scope of the paper and further studies are warranted.

These study findings are important given that iron deficiency, which is an important cause of anemia in arthritis patients, is partially preventable. Although not proven, the upper GI complications associated with nonselective NSAID use, including ulceration, perforation, and bleeding, could contribute to iron deficiency anemia in arthritis patients using these drugs [15,16,40]. Lower GI events are also an important contributor to safety throughout the entire GI tract [41-43] and may contribute to anemia, although there is no literature in anemic patients per se. Our data showing that anemia increases health care utilization in patients with arthritis suggest that treatment options should be examined carefully with consideration of the complete patient profile.

## Conclusion

In conclusion, the study hypothesis was confirmed in French public hospital settings; a clear difference in health care resource use attributable to anemia, adjusting for five confounders, exists in public hospitalizations. In private hospital settings, the additional resource use due to anemia was not clearly shown. This was probably due to the fact that patients seen in private hospitals have a less severe condition than those in public settings. These French database analyses provide some of the first evidence of the health care utilization and costs related to anemia specifically in patients with arthritis, and can be considered as a measure for the clinical significance of anemia. Overall, these findings warrant closer consideration of anemic arthritis patients in the clinical setting, and further research to better clarify the impact of anemia in populations with arthritis. Analysis of hospital databases in other European and non-European countries would enhance the available evidence regarding the impact of anemia in patients with OA and/or RA.

## Competing interests

R. Diazaraque, G. Zlateva, and L. Niculescu are full-time employees of Pfizer Inc. M. Viala-Danten is a full-time employee of Mapi Values, France, who were paid consultants to Pfizer in connection with this research. This study was funded by Pfizer Inc.

## Authors' contributions

GZ contributed to the data analysis, interpretation and writing of the manuscript; RD contributed to the data interpretation and writing of the manuscript; MVD contributed to the statistical analysis, interpretation and writing of the manuscript; LN contributed to the study design, analysis, interpretation and writing of the manuscript. The manuscript has not been submitted or is not simultaneously being submitted elsewhere, and all authors have read and approved the final version of the manuscript.

## Pre-publication history

The pre-publication history for this paper can be accessed here:

http://www.biomedcentral.com/1471-2318/10/59/prepub

## References

[B1] Helmick CG Felson DT Lawrence RC Gabriel S Hirsch R Kwoh CK Liang MH Kremers HM Mayes MD Merkel PA Pillemer SR Reveille JD Stone JH National Arthritis Data Workgroup Part I. Arthritis Rheum 2008 58 1 15 25 Estimates of the prevalence of arthritis and other rheumatic conditions in the United States 10.1002/art.23177 18163481

[B2] Brooks PM Impact of osteoarthritis on individuals and society: how much disability? Social consequences and health economic implications Curr Opin Rheumatol 2002 14 5 573 577 10.1097/00002281-200209000-00017 12192258

[B3] Gupta S Hawker GA Laporte A Croxford R Coyte PC The economic burden of disabling hip and knee osteoarthritis (OA) from the perspective of individuals living with this condition Rheumatology (Oxford) 2005 44 12 1531 1537 10.1093/rheumatology/kei049 16091394

[B4] Gabriel SE Crowson CS Campion ME O'Fallon WM Direct medical costs unique to people with arthritis J Rheumatol 1997 24 4 719 725 9101508

[B5] World Health Organization and the Bone and Joint Decade 2000-2010 The Burden of Musculoskeletal Conditions at the Start of the New Millennium Report of a WHO Scientific Group 2003 WHO Technical Report Series No.919. Geneva, Switzerland http://whqlibdoc.who.int/trs/WHO_TRS_919.pdf Accessed 2 Feb 2009 14679827

[B6] Kvien TK Epidemiology and burden of illness of rheumatoid arthritis Pharmacoeconomics 2004 22 2 Suppl 1 12 15157000 10.2165/00019053-200422001-00002

[B7] Cooper NJ Economic burden of rheumatoid arthritis: a systematic review Rheumatology (Oxford) 2000 39 1 28 33 10.1093/rheumatology/39.1.28 10662870

[B8] Guillemin F Durieux S Daures JP Lafuma A Saraux A Sibilia J Bourgeois P Sany J Costs of rheumatoid arthritis in France: a multicenter study of 1109 patients managed by hospital-based rheumatologists J Rheumatol 2004 31 7 1297 1304 15229947

[B9] World Health Organization Nutritional Anaemias Report of a WHO Scientific Group 1968 WHO Technical Report Series No.405, Geneva Switzerland, http://libdoc.who.int/trs/WHO_TRS_405.pdf Accessed 10 Dec 2008 4975372

[B10] Spence RK Medical and economic impact of anaemia in hospitalized patients Am J Health Syst Pharm 2007 64 16 Suppl 11 S3 10 10.2146/ajhp070244 17687068

[B11] Baer AN Dessypris EN Goldwasser E Krantz SB Blunted erythropoietin response to anaemia in rheumatoid arthritis Br J Haematol 1987 66 4 559 564 10.1111/j.1365-2141.1987.tb01344.x 3663512

[B12] Wilson A Yu HT Goodnough LT Nissenson AR Prevalence and outcomes of anemia in rheumatoid arthritis: a systematic review of the literature Am J Med 2004 116 Suppl 7A 50S 57S 10.1016/j.amjmed.2003.12.012 15050886

[B13] Hochberg MC Arnold CM Hogans BB Spivak JL Serum immunoreactive erythropoietin in rheumatoid arthritis: impaired response to anemia Arthritis Rheum 1988 31 10 1318 1321 10.1002/art.1780311016 3178910

[B14] Peeters HR Jongen-Lavrencic M Raja AN Ramdin HS Vreugdenhil G Breedveld FC Swaak AJ Course and characteristics of anaemia in patients with rheumatoid arthritis of recent onset Ann Rheum Dis 1996 55 3 162 168 10.1136/ard.55.3.162 8712878 PMC1010122

[B15] Baer AN Dessypris EN Krantz SB The pathogenesis of anemia in rheumatoid arthritis: a clinical and laboratory analysis Semin Arthritis Rheum 1990 19 4 209 223 10.1016/0049-0172(90)90001-V 2181669

[B16] Hawkey CJ NSAIDs, coxibs, and the intestine J Cardiovasc Pharmacol 2006 47 Suppl 1 S72 S75 10.1097/00005344-200605001-00013 16785834

[B17] Weiss G Goodnough LT Anemia of chronic disease N Engl J Med 2005 352 10 1011 1023 10.1056/NEJMra041809 15758012

[B18] Nissenson AR Goodnough LT Dubois RW Anemia: not just an innocent bystander? Arch Intern Med 2003 163 12 1400 1404 10.1001/archinte.163.12.1400 12824088

[B19] Gaskell H Derry S Andrew MR McQuay HJ Prevalence of anaemia in older persons: systematic review BMC Geriatr 2008 8 1 10.1186/1471-2318-8-1 18194534 PMC2248585

[B20] American Pain Society Guideline for the management of pain in osteoarthritis, rheumatoid arthritis, and juvenile chronic arthritis 2002 American Pain Society, Glenview, IL http://www.ampainsoc.org/pub/arthritis.htm Accessed 18 Aug 2010

[B21] Lawrence RC Helmick CG Arnett FC Deyo RA Felson DT Giannini EH Heyse SP Hirsch R Hochberg MC Hunder GG Liang MH Pillemer SR Steen VD Wolfe F Estimates of the prevalence of arthritis and selected musculoskeletal disorders in the United States Arthritis Rheum 1998 41 5 778 99 10.1002/1529-0131(199805)41:5<778::AID-ART4>3.0.CO;2-V 9588729

[B22] Arthritis Foundation Rheumatoid Arthritis: Who is at Risk? http://www.arthritis.org/disease-center.php?df=whos_at_risk&disease_id=31 Accessed 18 Aug 2010

[B23] Sharma JB Studd J Nutritional anaemia during pregnancy in non-industrialised countries Progress in Obstetrics and Gynaecology 2003 Edinburgh Churchill Livingstone 103 122

[B24] Murphy EA Bell AL Wojtulewski J Brzeski M Madhok R Capell HA Study of erythropoietin in treatment of anaemia in patients with rheumatoid arthritis BMJ 1994 309 6965 1337 1338 7866082 10.1136/bmj.309.6965.1337 PMC2541893

[B25] Tanaka N Ito K Ishii S Yamazaki I Autologous blood transfusion with recombinant erythropoietin treatment in anaemic patients with rheumatoid arthritis Clin Rheumatol 1999 18 4 293 298 10.1007/s100670050104 10468168

[B26] Anand IS Chandrashekhar Y Ferrari R Poole-Wilson PA Harris PC Pathogenesis of oedema in chronic severe anaemia: studies of body water and sodium, renal function, haemodynamic variables, and plasma hormones Br Heart J 1993 70 4 357 362 10.1136/hrt.70.4.357 8217445 PMC1025332

[B27] Penninx BW Pahor M Woodman RC Guralnik JM Anemia in old age is associated with increased mortality and hospitalization J Gerontol A Biol Sci Med Sci 2006 61 5 474 479 16720744 10.1093/gerona/61.5.474

[B28] Penninx BW Guralnik JM Onder G Ferrucci L Wallace RB Pahor M Anemia and decline in physical performance among older persons Am J Med 2003 115 2 104 110 10.1016/S0002-9343(03)00263-8 12893395

[B29] Penninx BW Pahor M Cesari M Corsi AM Woodman RC Bandinelli S Guralnik JM Ferrucci L Anemia is associated with disability and decreased physical performance and muscle strength in the elderly J Am Geriatr Soc 2004 52 5 719 724 10.1111/j.1532-5415.2004.52208.x 15086651

[B30] Penninx BW Pluijm SM Lips P Woodman R Miedema K Guralnik JM Deeg DJ Late-life anemia is associated with increased risk of recurrent falls J Am Geriatr Soc 2005 53 12 2106 2111 10.1111/j.1532-5415.2005.00491.x 16398894

[B31] Chaves PH Semba RD Leng SX Woodman RC Ferrucci L Guralnik JM Fried LP Impact of anemia and cardiovascular disease on frailty status of community-dwelling older women: the Women's Health and Aging Studies I and II J Gerontol A Biol Sci Med Sci 2005 60 6 729 735 15983175 10.1093/gerona/60.6.729

[B32] Chaves PHM Mody SH Blasi MV Siegartel LR Stem LS Doyle JJ Woodman RC Healthcare costs and resource utilization associated with chronic anemia in older adults J Manag Care Med 2005 8 13 20

[B33] Ershler WB Chen K Reyes EB DuBois R Economic burden of patients with anemia in selected diseases Value Health 2005 8 6 629 638 16283863 10.1111/j.1524-4733.2005.00058.x

[B34] Nissenson AR Wade S Goodnough T Knight K Dubois RW Economic burden of anemia in an insured population J Manag Care Pharm 2005 11 7 565 574 16137214 10.18553/jmcp.2005.11.7.565 PMC10437330

[B35] College des Economistes de la Sante (CES) Guide Methodologique pour l'evaluation economique des Strategies de Sante http://www.rees-france.com/article.php3?id_article=126 Accessed 18 Aug 2010

[B36] World Health Organization International Classification of Diseases (ICD) http://www.who.int/classifications/icd/en/Accessed 18 Aug 2010

[B37] Echelle Nationale des couts de reference par GHM 2002 (hospitalisations publiques 2001) Disponible aupres du PMSI/ATIH 2002

[B38] Echelle des couts prives issus des facturations par GHM 2001 Disponible aupres de PMSI/ATIH 2001

[B39] Assaf AR Lapane KL McKenney JL Carleton RA Possible influence of the prospective payment system on the assignment of discharge diagnoses for coronary heart disease N Engl J Med 1993 329 13 931 935 10.1056/NEJM199309233291307 8361508

[B40] Segal R Baumoehl Y Elkayam O Levartovsky D Litinsky I Paran D Wigler I Habot B Leibovitz A Sela BA Caspi D Anemia, serum vitamin B12, and folic acid in patients with rheumatoid arthritis, psoriatic arthritis, and systemic lupus erythematosus Rheumatol Int 2004 24 1 14 19 10.1007/s00296-003-0323-2 12720045

[B41] Chan FK Hung LC Suen BY Wu JC Lee KC Leung VK Hui AJ To KF Leung WK Wong VW Chung SC Sung JJ Celecoxib versus diclofenac and omeprazole in reducing the risk of recurrent ulcer bleeding in patients with arthritis N Engl J Med 2002 347 26 2104 2110 10.1056/NEJMoa021907 12501222

[B42] Lanas A Garcia-Rodriguez LA Ponce M Rodrigo L Bujanda L Gilbert JP Alonso-Abreu I Castro-Fernandez M Perez Aisa A Polo-Tomas MP Calvet X Garcia S Clinical impact and time trends of upper and lower gastrointestinal complications [abstract] Gastroenterology 2008 134 4 A18 10.1016/S0016-5085(08)60096-7

[B43] Laine L Connors LG Reicin A Hawkey CJ Burgos-Vargas R Schnitzer TJ Yu Q Bombardier C Serious lower gastrointestinal clinical events with nonselective NSAID or coxib use Gastroenterology 2003 124 2 288 292 10.1053/gast.2003.50054 12557133

